# Molecular basis for specificity of the Met1-linked polyubiquitin signal

**DOI:** 10.1042/BST20160227

**Published:** 2016-12-02

**Authors:** Paul R. Elliott

**Affiliations:** MRC Laboratory of Molecular Biology, Francis Crick Avenue, Cambridge Biomedical Campus, Cambridge CB2 0QH, U.K.

**Keywords:** inflammation, post-translational modification, ubiquitin signalling

## Abstract

The post-translational modification of proteins provides a rapid and versatile system for regulating all signalling pathways. Protein ubiquitination is one such type of post-translational modification involved in controlling numerous cellular processes. The unique ability of ubiquitin to form polyubiquitin chains creates a highly complex code responsible for different subsequent signalling outcomes. Specialised enzymes (‘writers’) generate the ubiquitin code, whereas other enzymes (‘erasers’) disassemble it. Importantly, the ubiquitin code is deciphered by different ubiquitin-binding proteins (‘readers’) functioning to elicit particular cellular responses. Ten years ago, the methionine1 (Met1)-linked (linear) polyubiquitin code was first identified and the intervening years have witnessed a seismic shift in our understanding of Met1-linked polyubiquitin in cellular processes, particularly inflammatory signalling. This review will discuss the molecular mechanisms of specificity determination within Met1-linked polyubiquitin signalling.

## Introduction

Post-translational modification of proteins is one mechanism by which signalling cascades, such as those activating nuclear factor-κB (NF-κB) and mitogen-activated protein kinases, are regulated. This serves to regulate protein substrate recruitment, activation or inactivation. Over 200 types of post-translational modifications exist [1,2]. One such modification is the covalent attachment of the small (76 amino acid) protein ubiquitin onto the ε-amino group of a substrate lysine (Lys).

### The ubiquitin code

**Figure 1.**
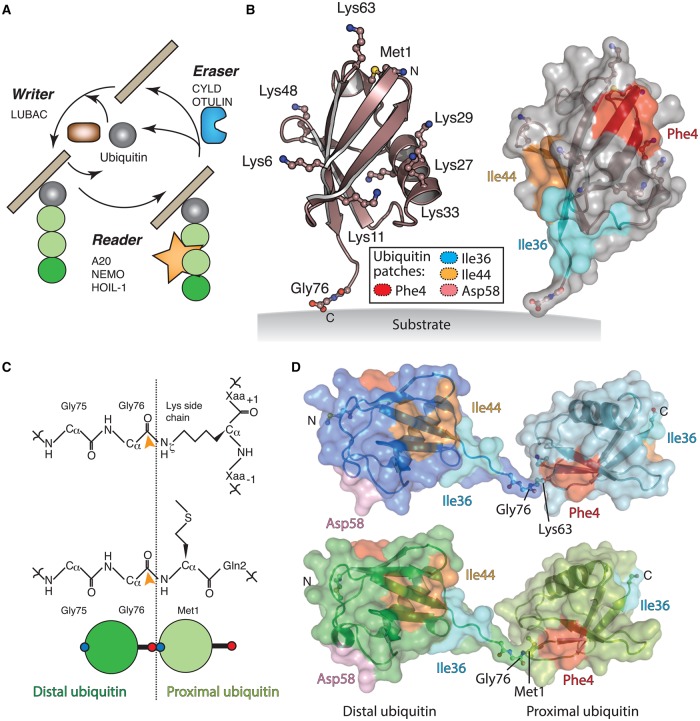
The nature of the Met1-linked polyubiquitin code. (**A**) The Met1-linked polyubiquitin code is generated by the writer, LUBAC (brown); interpreted by readers such as A20, NEMO and HOIL-1 (orange) and erased by DUBs CYLD and OTULIN (blue). A substrate is depicted as a grey bar. (**B**) A single ubiquitin moiety is shown as cartoon with the side chain lysine residues and the amino-terminus of Met1 shown in ball-and-stick format. Additionally, the C-terminal glycine residue is shown in the ball-and-stick format. Right, the surface of ubiquitin is shown with hydrophobic patches coloured: Phe4 patch, red; Ile36 patch, cyan; Ile44 patch, orange. (**C**) Chemical representation of the isopeptide bond; middle, chemical representation of the peptide bond for a Met1 linkage; bottom, schematic representation of Met1-linked diubiquitin. The distal moiety is shown in dark green and the proximal moiety in light green. The amino-terminus (Met1) is shown as a blue circle and the carboxy-terminus is shown as red circles. (**D**) Surface representation of Lys63-linked diubiquitin (top, blue) and Met1-linked diubiquitin (bottom, green) viewed in the same orientation. The hydrophobic patches are shown on each ubiquitin moiety. The peptide linkage (Met1) and isopeptide (Lys63) linkages are shown, as are the Met1 amino-terminus on the distal moiety (N) and carboxy-terminus on the proximal ubiquitin moiety (C).

Ubiquitin is a highly versatile post-translational modification. Dedicated ‘writers’ assemble the code, ‘readers’ decipher the code, and ‘erasers’ reverse the code (Figure 1A). Ubiquitin is attached to substrates through an ATP-dependent mechanism involving a sequential cascade of ubiquitin-activating (E1) and ubiquitin-conjugating (E2) enzymes and ubiquitin ligases (E3), resulting in the transfer of ubiquitin onto the ε-amino group of a substrate lysine forming an isopeptide bond [3]. The reverse of this reaction is catalysed by deubiquitinating enzymes (DUBs) [4,5]. Dedicated ubiquitin-binding domains (UBDs) are capable of deciphering the ubiquitin code and eliciting an appropriate response [6].

Perhaps with the exception of glycosylation, the ubiquitin code represents one of the most complex types of post-translational modifications, as protein substrates can be modified by a single ubiquitin moiety on one or multiple lysine residues resulting in mono-/multi-monoubiquitination. However, further complexity arises since ubiquitin itself can be ubiquitinated, resulting in the formation of polyubiquitin chains. The seven internal lysine residues of ubiquitin (Lys6, Lys11, Lys27, Lys29, Lys33, Lys48, and Lys63) all serve as points for ubiquitination. In addition to the lysine side chains, the α-amino-terminus of methionine1 (Met1) is a donor for additional ubiquitin attachment, forming Met1-linked (linear) polyubiquitin chains. Thus, eight types of homotypic chains (chains of one linkage type) can be generated (Figure 1B).

An added layer of complexity is achieved through heterotypic (branched) chains, in which a single ubiquitin moiety is ubiquitinated at two or more different lysine residues. Details of the biological importance of branched chains are emerging and show that branched chains play an important role in cellular processes. For example, branched Lys11/Lys48 chains generated by anaphase-promoting complex (APC/C) are important for cell cycle progression [7] and branched Lys11/Lys63 chains are important for major histocompatibility complex (MHC) class 1 endocytosis [8]. Additionally, branched Lys63/Met1 chains have been shown to be important during interleukin-1β (IL-1β) signalling [9] and are discussed in more detail later.

Proteomic analysis has revealed that all eight types of polyubiquitin chains exist within cells and potentially encode for different signalling outcomes [10,11]. Lys48-linked polyubiquitin chains serve as a signal for substrate degradation by the 26S proteasome [12], while the Lys11-linked signal is implicated in proteasomal degradation of substrates within the cell cycle [13]. Additionally, Lys63-linked chains serve as non-degradative signals in many signalling processes [14]. Roles of the atypical chains (Lys6, Lys27, Lys29, and Lys33) remain less well characterised, but are slowly emerging as important signals in particular cellular processes (reviewed in ref. [15]).

This review focuses on the other atypical chain type, Met1-linked polyubiquitin chains, and their involvement in inflammatory signalling processes. An important feature regarding Met1-linked polyubiquitin chains is the chemical nature of the peptide linkage between the C-terminus of one ubiquitin (distal) moiety and the Met1 amino-terminus of the second ubiquitin (proximal) moiety (Figure 1C). Isopeptide linkages have the potential for greater flexibility between the two ubiquitin moieties, through increased rotation about the lysine side chain (Figure 1C). Additionally, the proximal Met1 side chain needs to be accommodated into the active site of any DUB that is to cleave this linkage type.

Interestingly, Lys63 and Met1 linkages have similar topologies owing to the attachment points of the Lys63 ε-amino group and the α-amino-terminus of Met1 being only 7 Å apart (Figure 1D). Importantly, Lys63- and Met1-specific UBDs and DUBs have evolved different mechanisms to distinguish between the two linkage types.

### Deciphering the ubiquitin code

The fact that all eight ubiquitin linkages code for defined biological roles suggests that different proteins can distinguish one linkage type from another. This raises the question: what is the distinction between different linkage types? The answer lies in the remarkable ability of ubiquitin to participate in numerous non-covalent interactions via distinct hydrophobic patches (Figure 1B). For example, the Ile44 patch (Leu8, Ile44, His68, and Val70) is the canonical site for binding UBDs [16]. Additionally, the Ile36 (Leu8, Ile36, Leu71, and Leu73) and the Phe4 (Gln2, Phe4, and Thr14) patches are bound by UBDs and DUBs [17–19].

Furthermore, the Asp58 patch (Arg54, Thr55, Ser57, and Asp58) is located on the opposite face of ubiquitin and, unlike the other patches, mediates only polar contacts. To date, only the ubiquitin-binding zinc (Zn) finger (UBZ) domain of Rabex5 utilises this patch [20]. Importantly, the nature of the linkage between the distal and proximal ubiquitin moieties (for example Lys48 versus Lys63) will define which patches are presented and recognised by linkage-selective UBDs and DUBs.

### Ubiquitin in inflammatory signalling

The inflammatory pathway is a broad term used to describe several pathways that, once stimulated, result in specialised signalling cascades culminating in activation of transcription factors such as NF-κB that drive transcription of genes involved in the orchestration of inflammation. These transcription factors regulate a variety of immune responses through expression of cytokines, chemokines and pro-inflammatory and survival genes (reviewed in ref. [21]). Aberrant inflammatory signalling is the cause of many human inflammatory and autoimmune diseases, obesity and certain cancers [21].

**Figure 2.**
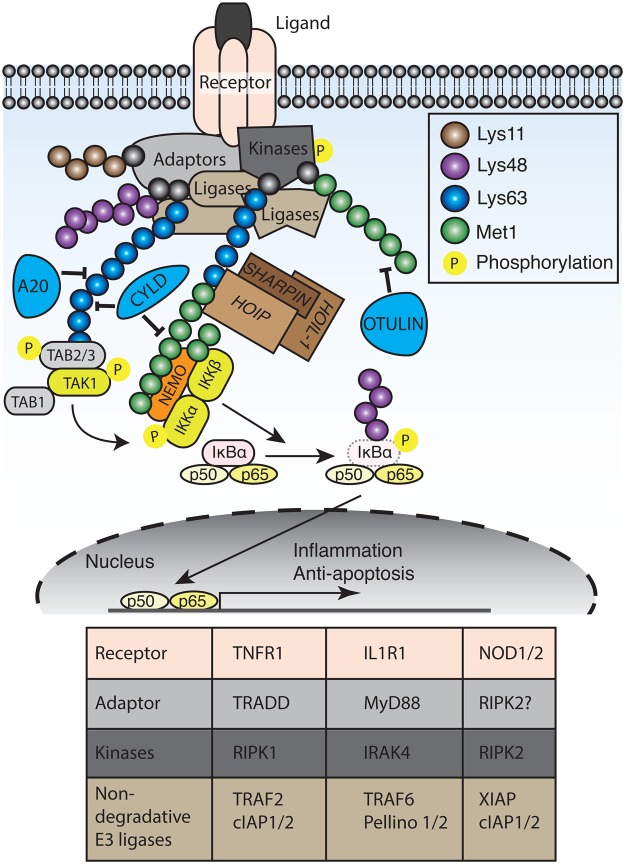
Met1-linked polyubiquitin signalling in inflammatory pathways. An extracellular receptor is shown (but could equally apply to intracellular receptors such as NOD1/2). Upon receptor activation, different adaptors and kinases are recruited. In addition, E3 ubiquitin ligases are recruited, generating non-degradative polyubiquitin chains that serve to recruit other kinases and adaptors (notably TAB2/3 TAK1 and the IKK complex). LUBAC is also recruited, which generates Met1-linked polyubiquitin chains. Activated IKK phosphorylates IκBα, resulting in its concurrent polyubiquitination and degradation by the proteasome, releasing NF-κB, which activates pro-inflammatory and anti-apoptosis genes. Various DUBs, such as A20, CYLD and OTULIN, regulate the polyubiquitin signal. The table lists the known adaptors, kinases, and non-degradative ubiquitin ligases recruited to the three most-studied inflammatory receptor complexes.

Although the stimulant and activated receptors vary, inflammatory signalling cascades share a common architecture (Figure 2). Activated receptors recruit adaptors, such as myeloid differentiation primary response gene 88 (MyD88) and tumour necrosis factor (TNF) receptor 1 (TNFR1)-associated death domain (TRADD); kinases, for example receptor-interacting protein kinase 1 (RIPK1) and IL-1 receptor-associated kinase 4 (IRAK4); and E3 ubiquitin ligases, such as TNF receptor-associated factor 6 (TRAF6) and cellular inhibitor of apoptosis 1/2 (cIAP1/2). These ligases generate Lys63- and Lys11-linked polyubiquitin chains that serve as recruitment signals for further kinases and adaptors, including the transforming growth factor-β-activated kinase 1 (TAK1)-binding protein (TAB)2/3 complex and the inhibitor of κB (IκB) kinase (IKK) complex (Figure 2). Recruitment of the IKK complex [composed of kinases: IKKα, IKKβ, and the adaptor, IKKγ, also known as nuclear factor-κB essential modifier (NEMO)] results in IκB phosphorylation, leading to its Lys48-linked polyubiquitination and subsequent proteasomal degradation. NF-κB, normally sequestered in the cytosol by IκB, is thereby free to translocate to the nucleus and drive gene transcription (Figure 2).

In addition to Lys48-linked polyubiquitin chains, other linkages have been demonstrated as crucial for effective NF-κB activation (reviewed in ref. [22,23]). An elegant ubiquitin replacement strategy by Chen and co-workers first identified the importance of Lys63 linkages, mediated by TRAF6 [24]. Furthermore, Lys63 polyubiquitin chains activate TAK1 through binding TAB2/3 [25] and even unanchored Lys63 linkages (those not attached to substrates) are capable of activating TAK1 [26]. Subsequently, cIAP1/2 has been shown to synthesise Lys11, Lys48, and Lys63-linked polyubiquitin chains onto RIPK1 [27–29]. The additional identification of the linear ubiquitin chain assembly complex (LUBAC) by Iwai and co-workers [30] and the roles of Met1 linkages in NF-κB-mediated signalling [31] have added an extra dimension to non-degradative polyubiquitin chains in inflammatory signalling (Figure 2) (reviewed in ref. [22,32]).

### Biological roles of Met1 chains

Until a decade ago, Met1-linked polyubiquitin chains were thought to only exist as a product of translation of the polyubiquitin genes *UBB* and *UBC* maintaining ubiquitin homeostasis. The identification of LUBAC by Iwai and co-workers [30], and the demonstration that Met1-linked polyubiquitin chains are generated as a signalling molecule, turned this notion on its head and paved the way for a series of transformative discoveries, particularly in the understanding of inflammatory pathways, in which Met1 linkages and other ubiquitin linkages are crucial for mediating appropriate cellular responses.

**Figure 3.**
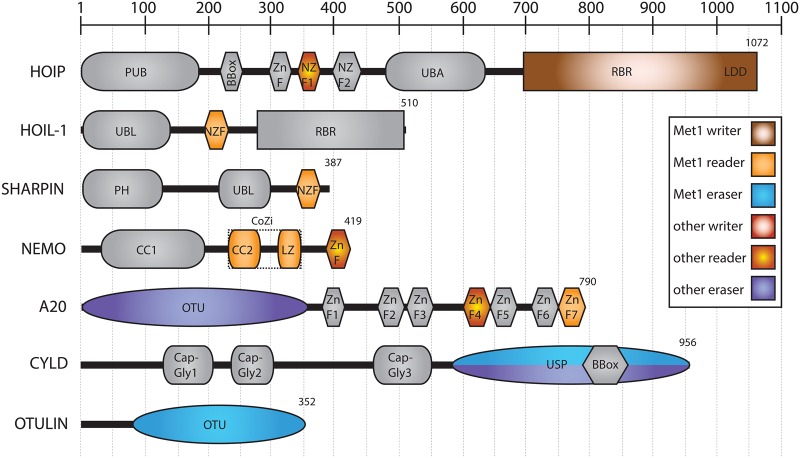
Proteins that regulate the Met1 code. Schematic representation of the proteins and enzymes that regulate the Met1-linked polyubiquitin code with domains drawn relative to one another and domain boundaries indicated. Domains that are involved in regulating polyubiquitin signalling are coloured accordingly.

LUBAC is composed of three proteins: haem-oxidised iron-responsive element-binding protein 2 (IRP2) ubiquitin ligase-1 (HOIL-1), HOIL-1-interacting protein (HOIP), and SHANK-associated RH domain interactor (SHARPIN) [31,33–35] (Figure 3). HOIP is the catalytic component of LUBAC and, like HOIL-1, belongs to the really interesting new gene (RING) in-between RING (RBR) family of E3 ubiquitin ligases (see below). Mouse knockout studies have demonstrated the importance of LUBAC as an important mediator of NF-κB signalling pathways [33–36], as have discoveries of inherited genetic mutations of LUBAC in patients [37,38].

Met1-linked polyubiquitin linkages have subsequently been shown to be important in most innate immune receptor complexes (reviewed in ref. [32]), including the TNFR1; nucleotide-binding and oligomerisation domain-containing proteins (NOD)1 and NOD2; retinoic acid-inducible gene 1; IL-1β; toll-like receptors and CD40 and B-cell receptors (Figure 2). Importantly, Lys63 linkages, generated by cIAP1/2 [31,39], X-linked inhibitor of apoptosis protein (XIAP) [40], TRAF6 or Pellino 2 [9], recruit the TAB1-TAK1-TAB2/3 kinase complex and LUBAC. LUBAC recruitment and concurrent Met1-linked polyubiquitination of substrates results in NEMO recruitment and activation of IKKα and IKKβ, resulting in IκB phosphorylation and degradation.

Although the above pathways all utilise Lys63 and Met1 linkages to achieve effective NF-κB signalling, some pathways are more dependent on particular linkage types than others. It has been elegantly shown that Lys63 linkages are dispensable for TNFR1 signalling but crucial for IL-1β signalling [41]. However, while Met1 linkages are essential for IL-1β signalling [37,38], and for NOD1 and NOD2 signalling [40,42,43], Met1 linkages were not thought to be essential for TNFR1 signalling [39,44] and B-cell receptor-mediated NF-κB activation [44]. However, regarding B-cell receptor signalling, rare single-nucleotide polymorphisms in the *RNF31* gene, resulting in hyperactive LUBAC, are important for driving B-cell receptor signalling in diffuse large B cell lymphomas [45]. Additionally, Lys63 linkages generated by cIAP are present at the CARMA1–BCL10–MALT1 (CBM) complex, which recruits LUBAC [46].

The regulation of the Met1 signal is defined by the ‘writer’ LUBAC, which generates Met1-linked polyubiquitin chains; ‘readers’ such as NEMO that detect Met1 linkages; and ‘erasers’ such as the DUBs cylindromatosis tumour suppressor (CYLD) and OTU domain deubiquitinase with LINear linkage specificity (OTULIN; Figure 3). Notably, HOIP and OTULIN are specific for assembling and hydrolysing Met1 linkages, respectively. Some of the other proteins involved in Met1-linked signalling have overlapping roles regulating other parts of the ubiquitin code, notably A20 (Lys48, Lys63, and Met1) and CYLD (Lys63 and Met1). This reflects the presence of other ubiquitin linkages in signalling cascades, particularly in inflammatory signalling. Below, I describe how the ‘writers’, ‘readers’, and ‘erasers’ of the Met1 code achieve specificity at the molecular level.

## Molecular basis for Met1 specificity

In comparison with knowledge of other ubiquitin signals, the Met1 signal is unique in that the enzymes responsible for the assembly, recognition, and disassembly are known. The following sections describe the molecular details for how each of these components achieves specificity towards regulating the Met1 signal.

### The writer: LUBAC

Both HOIP and HOIL-1 belong to the RING-in-between-RING (RBR) family of E3 ubiquitin ligases [47]. Thirteen human RBR enzymes have been identified in the human genome and include the E3 ubiquitin ligase Parkin, frequently mutated in autosomal recessive juvenile Parkinsonism. In contrast with the RING or homologous to the E6-AP C-terminus (HECT) family of E3 ubiquitin ligases that transfer ubiquitin from the charged E2 onto substrates via a scaffold or directly through the E3 (RING and HECT, respectively), RBRs transfer ubiquitin from a charged E2 onto the substrate through a RING/HECT hybrid mechanism and are the third and smallest class of E3 ubiquitin ligases [48,49].

### Achieving HOIP specificity: linear ubiquitin chain determination domain

HOIP is the only known ubiquitin ligase possessing the ability to assemble Met1-linked polyubiquitin chains. Biochemical analysis of HOIP from the Rittinger and Sixma laboratories demonstrated that HOIP contains an additional domain, C-terminal to the RBR, termed the linear ubiquitin chain determination domain (LDD) [50,51].

**Figure 4.**
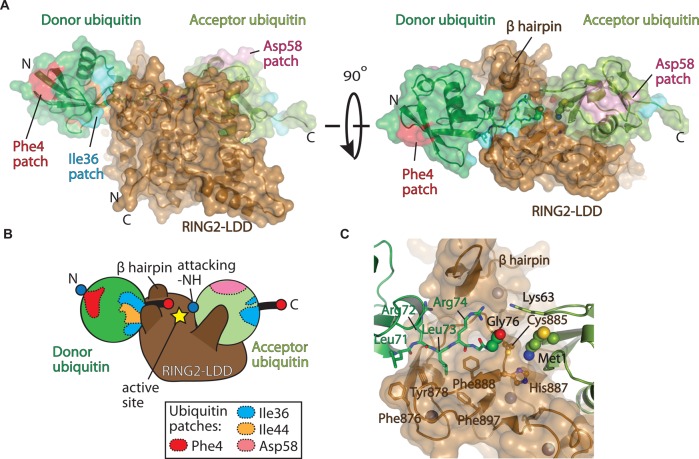
Molecular basis of Met1 assembly by HOIP (‘writer’). (**A**) Structure of the HOIP RING2–LDD (brown) bound to ubiquitin (PDB ID: 4LJO). The donor and acceptor ubiquitin moieties are shown as surfaces and coloured dark and light green, respectively. Ubiquitin patches as described in Figure 1B are shown. (**B**) Cartoon representation of **A** showing the regions of ubiquitin engaged by the RING2–LDD. (**C**) Close-up view showing the cradling of the donor and acceptor ubiquitin moieties by the RING2–LDD (brown surface). The donor ubiquitin C-terminus and acceptor ubiquitin N-terminus are shown as balls. The catalytic cysteine (Cys885) and histidine (H887) residues are shown in ball-and-stick representation.

The structure of the RING2–LDD in complex with non-covalent monoubiquitin revealed how HOIP is capable of achieving specific assembly of Met1-linked polyubiquitin chains [52]. Unexpectedly, the LDD forms an extension of the RING2 domain (Figure 4A). HOIP RING2–LDD comprises a seven-member helical scaffold with two Zn finger (ZnF) insertions. The majority of the interactions between the RING2–LDD and the donor ubiquitin moiety centre around the Ile36 patch and the C-terminus of ubiquitin. Here, the RING2 forms hydrophobic interactions along the ubiquitin C-terminal tail, positioning the tail in an elongated conformation. The hydrophobic residues from the RING2 are conserved among other RBR family members and may represent a general model for the positioning of the donor ubiquitin [52]. Additionally, a β-hairpin, not found in other members of the RBR family, forms polar and salt-bridge interactions with the C-terminal tail of the donor ubiquitin, further stabilising the elongated conformation (Figure 4B). The cradling of the donor ubiquitin C-terminus in an extended conformation appears to be a general mechanism of ubiquitin transamidation, as this extended conformation has been observed in RING E2∼ubiquitin complexes [53–56] and more recently in HECT E3 ubiquitin ligases [57,58].

The acceptor ubiquitin is orientated with the α-amino-terminus of Met1 positioned 6.5 Å from the C-terminus of the donor ubiquitin and 3.5 Å from the RING2 catalytic cysteine (Cys885), orientated for nucleophilic attack and peptide bond formation (Figure 4C). Extensive contacts from the RING2 and LDD orientate the acceptor ubiquitin in such a way that only the α-amino-terminus of Met1 is positioned for attack and that no other ε-amino groups from one of the seven side chain lysine residues of the acceptor ubiquitin are in close proximity. Thus, Met1 specificity is achieved.

With the exception of a few polar contacts between the LDD and the Phe4 patch, most interactions from the LDD form complementary polar and salt-bridge interactions with the amphipathic acceptor ubiquitin α helix, with additional interactions from the LDD to the base of the ubiquitin α helix. Interestingly, the Parkin RING2 does not contain a C-terminal extension such as the LDD. Thus, it will be interesting to determine how other RBRs achieve linkage specificity.

Mechanistically, the structure of the HOIP RING2–LDD in complex with ubiquitin revealed a catalytic histidine (His887) in proximity to the catalytic cysteine and the acceptor ubiquitin α-amino-terminus. His887 is not required for transthiolation (transfer of the donor ubiquitin from the E2∼ubiquitin to HOIP Cys885), but it is important for diubiquitin formation. Thus, His887 may act as a general base to increase the nucleophilicity of the attacking Met1 α-amino-terminus from the acceptor ubiquitin moiety. Interestingly, His887 is not conserved in the RBR domain of HOIL-1 and, furthermore, HOIL-1 lacks other key residues for the function of the RBR domain, probably explaining its apparent lack of activity [51].

## Readers

The *raison d'être* of the ubiquitin code is to be deciphered. This is typically mediated by small UBDs. To date, the protein with the highest affinity for Met1 linkages is NEMO, where binding is crucial not only for recruitment of the IKK complex to the emerging signalling complex, but also for activating IKKα and IKKβ *in vitro*. In addition to NEMO, other proteins are known to bind Met1 linkages. Curiously, two components of LUBAC, HOIL-1 and SHARPIN, preferentially bind Met1 linkages (and will be discussed later). Additionally, it has been reported that cIAP1/2 and XIAP also bind to Met1 linkages [59], although conclusive biochemical evidence is currently lacking.

### Nuclear factor-κB essential modifier

NEMO contains two coiled coil (CC) regions (CC1 and CC2), a leucine zipper (LZ) region and a C-terminal ZnF domain (Figure 3). Initially, the mechanisms surrounding NEMO recruitment to inflammatory complexes were unclear, since full-length NEMO had been shown to bind to Met1 and Lys63 linkages with differing affinities [60]. In part, this can be ascribed to avidity effects of the C-terminal ZnF domain, which does not have any linkage preference [61].

**Figure 5.**
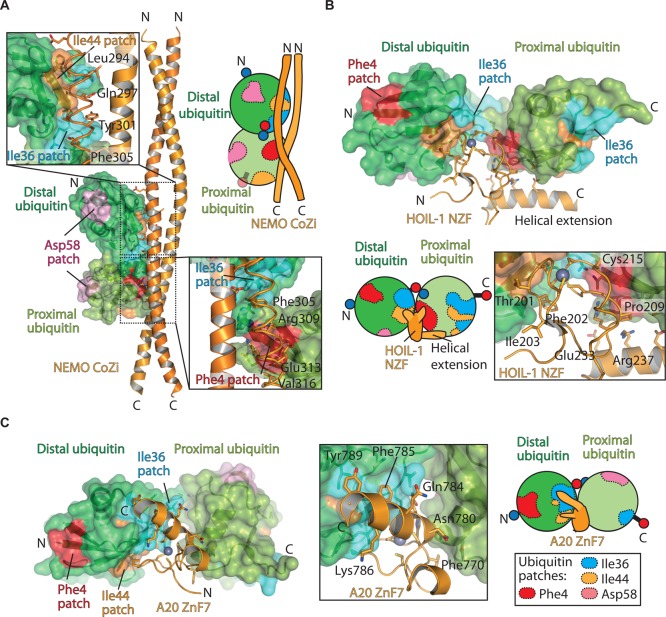
Molecular basis of Met1 recognition (‘readers’). (**A**) Structure of mouse NEMO CoZi domain (orange) bound to Met1 diubiquitin (green surface) (PDB ID: 2ZVN) with different ubiquitin patches highlighted as in Figure 1B. Insert, close-up view of both distal and proximal ubiquitin interactions. For clarity, one CoZi domain is shown as a cartoon, while the other domain is shown as a ribbon to highlight the side chains that interact with the ubiquitin moieties. (**B**) Structure of the HOIL-1 NZF bound to Met1-linked diubiquitin (PDB ID: 3B0A). The HOIL-1 NZF domain is shown as a cartoon (orange) with Met1 diubiquitin as a surface with different patches coloured. Insert, the Zn co-ordinating loop that engages with both distal and proximal ubiquitin moieties is shown. (**C**) Structure of A20 ZnF7 bound to Met1-linked diubiquitin (PDB ID: 3VUW). Insert, residues from A20 ZnF7 that interact with the distal and proximal ubiquitin moieties are shown.

Several groups have since clarified this discrepancy and demonstrated that NEMO binds Met1 linkages preferentially over Lys63 linkages [19,60,62–64]. In fact, the NEMO CC2 LZ (CoZi) domain binds Met1 linkages with low micromolar affinity (1.6 µM), some 100-fold greater than Lys63 linkages [64]. The structure of the NEMO CoZi domain in complex with Met1 diubiquitin revealed the molecular basis for this specificity [19]. The NEMO CoZi domain adopts a parallel coiled-coil conformation and binds two Met1-linked diubiquitins. The distal ubiquitin moiety binds through the canonical Ile44 patch. Backbone interactions between ubiquitin residues Leu73 and Arg74 and NEMO further stabilise the distal binding site. Interestingly, residues that form the Phe4 patch on the proximal ubiquitin moiety co-ordinate NEMO via extensive polar contacts (Figure 5A). Mutations in NEMO that prevent ubiquitin binding reduce the activation of the IKK complex [65,66].

The structure of NEMO CoZi in complex with Lys63-linked diubiquitin revealed why Lys63-linked chains bind with lower affinity: in this situation, only the distal ubiquitin moiety engages with NEMO, whereas the proximal ubiquitin moiety is unable to simultaneously bind [63]. Curiously, solution studies clearly show the stoichiometry of the NEMO CoZi domain to be 2:1 (one NEMO dimer binds one Met1-linked diubiquitin) [64,67], which cannot be explained by the crystal structure [19].

The mechanisms underlying IKK activation through Met1-linked polyubiquitin chain binding to NEMO are currently enigmatic; the IKK interaction region is at the N-terminus of NEMO, thus how can ubiquitin binding to the CoZi domain activate IKK? The structure of inhibitor-bound IKKβ from *Xenopus laevis* revealed that IKKβ contains a dimerisation domain common for the IKK-related family of kinases [68] and suggested that IKKβ oligomerisation may be required for activation. This is supported by another crystallographic and solution study [69]. One activation mechanism centres on a conformational change with slight unwinding of the coiled-coil upon Met1-linked diubiquitin binding to the CoZi domain, resulting in a conformational change propagated to the IKK-binding site, resulting in *trans*-activation of IKK [19]. Consistently, a constitutively active IKK is formed by a single-point mutation within the CoZi domain (K277A, human isoform) that stabilises the coiled-coil [70], suggesting long range allosteric activation of IKKβ. However, it should be noted that previous studies have shown that in TAK1-deficient cells neither TNF nor IL-1 are sufficient for NF-κB activation [71]. This suggests that although NEMO binding to Met1 linkages may be sufficient for IKK activation *in vitro*, phosphorylation of IKK by the TAK1 kinase complex is also required *in vivo*. This will be discussed further (see *The Emerging Roles of Branched Chains* section).

### HOIL-1 Npl4 zinc finger specificity

The nuclear protein localisation 4 (Npl4) zinc finger (NZF) domain of HOIL-1 binds specifically to Met1 linkages. NZF domains have been identified in over 100 proteins and, depending on the presence of a T-F/Y-x_n_-ϕ motif, are capable of binding to ubiquitin [72]. Other NZF domains have been shown to specifically bind different linkages: Trabid NZF1 specifically recognises Lys29 and Lys33 linkages [73,74], whereas TAB2 NZF recognises Lys63 linkages [75,76]. All NZF-binding modes known so far involve recognition of the distal ubiquitin moiety through the T-F/Y-x_n_-ϕ motif and do not recognise sites around the isopeptide linkage; however, the HOIL-1 NZF simultaneously engages with both distal and proximal ubiquitin moieties. HOIL-1 contains the canonical T-F/Y-x_n_-ϕ motif (Thr201–Phe202–Met213), which engages with the Ile44 patch of the distal ubiquitin moiety. Like the CoZi domain of NEMO discussed above, HOIL-1 NZF engages the Phe4 patch of the proximal ubiquitin moiety. Furthermore, HOIL-1 NZF contains a highly conserved C-terminal helical extension that engages with the proximal ubiquitin moiety and the Phe4 patch (Figure 5B). This C-terminal helix does not confer specificity, but rather enhances the affinity of the NZF domain for Met1 linkages by several-fold (*K*_D_ 17 µM).

In addition, SHARPIN has been shown to bind preferentially to Met1 linkages over Lys63 linkages [33]. This is probably explained by SHARPIN containing a C-terminal NZF domain and the equivalent residues that can bind to the Phe4 patch of the proximal ubiquitin moiety.

### A20 zinc finger 7 specificity

A20 is an important DUB that functions to regulate the NF-κB response [77]. A20 is a member of the ovarian tumour (OTU) family of DUBs and has been shown to display Lys48 cleavage *in vitro* [78]. Recently, A20 has been shown to undergo IKK-dependent phosphorylation that allows for Lys63-dependent cleavage *in vitro* and *in vivo* [79]. In addition to the OTU domain, A20 contains seven A20-like ZnF domains (Figure 3). The seven ZnF domains differ in function from one another, for example, ZnF1 binds RIPK1 [80], whereas ZnF4 binds monoubiquitin and also Lys63 linkages [80] and is thought to display ubiquitin ligase activity [81]. However, ZnF4 is also important for the indirect interaction with two E3 ubiquitin ligases, ITCH and RNF11 [82,83], which may explain the apparent ZnF4 E3 ubiquitin ligase activity. In contrast, ZnF7 binds Met1 linkages with 400-fold greater affinity than Lys63 linkages [79,84].

The structure of ZnF7 bound to Met1 diubiquitin and tetraubiquitin revealed that ZnF7 binds both distal and proximal ubiquitin moieties simultaneously [84]. Residues from the central ZnF helix form primarily hydrophobic interactions with the Ile36 patch of the distal ubiquitin moiety. The A20 Zn-co-ordinated loop inserts within a groove of the Ile44 patch of the distal moiety with additional contacts to the proximal ubiquitin moiety (Figure 5C). Importantly, residues that engage the distal ubiquitin moiety are conserved among ZnF7 orthologues, but are not conserved among ZnF1–ZnF6, suggesting that only ZnF7 has evolved Met1 specificity.

## Erasers

Precise regulation of the ubiquitin signal is crucial. This is achieved by the fine balance between the assembly of the ubiquitin code and DUBs, which serve to hydrolyse polyubiquitin chains. There are five DUB families (reviewed in refs [4,5]). Owing to the nature of the Met1 linkage, few DUBs are capable of effectively hydrolysing Met1 linkages. The exception is USP5 (isoT), which is the dedicated DUB for cleaving the gene product of polyubiquitin translation. USP5 contains a unique UBD that specifically recognises the free C-terminus of a ubiquitin chain [85]. As such, USP5 is capable of effectively cleaving unanchored Met1-linked polyubiquitin chains through *exo* activity, cleaving one ubiquitin moiety at a time from the proximal end.

However, with the identification of LUBAC and the specificity of defined readers, such as NEMO, the question remained whether there were any DUBs that could specifically hydolyse the Met1-linked polyubiquitin signal. The following section focuses on two DUBs: first, CYLD, which has been implicated in a variety of signalling pathways [86] and in particular is a negative regulator of the NF-κB response [87–89] and secondly, OTULIN, which specifically hydrolyses Met1 linkages [90,91].

### Cylindromatosis tumour suppressor

CYLD is a member of the ubiquitin-specific protease (USP) family of DUBs. CYLD contains three N-terminal cytoskeletal-associated protein-glycine-conserved (CAP-Gly) domains that mediate association with microtubules [92,93] and NEMO [88,94], and a C-terminal USP domain that contains an inserted B-Box [95] (Figure 3). Mutations within the USP domain of CYLD are the primary cause of familial cylindromatosis, the formation of tumours of the skin from hair follicles and sweat glands [96].

CYLD has been shown to process polyubiquitin chains as an *endo* DUB, cleaving within the polyubiquitin chain [95]. CYLD displays strong preference for Lys63 and Met1 linkages, with limited Lys48 cleavage observed *in vitro* [95]. In contrast, other USPs are isopeptidases and do not display linkage specificity, but are unable to cleave the peptide Met1 linkage as efficiently [97,98]. However, to date, only a handful of USPs have been fully characterised and it is likely that other USPs may display different activities through additional mechanisms, given the large number of accessory domains and insertions present within the catalytic USP domain [5,99].

**Figure 6.**
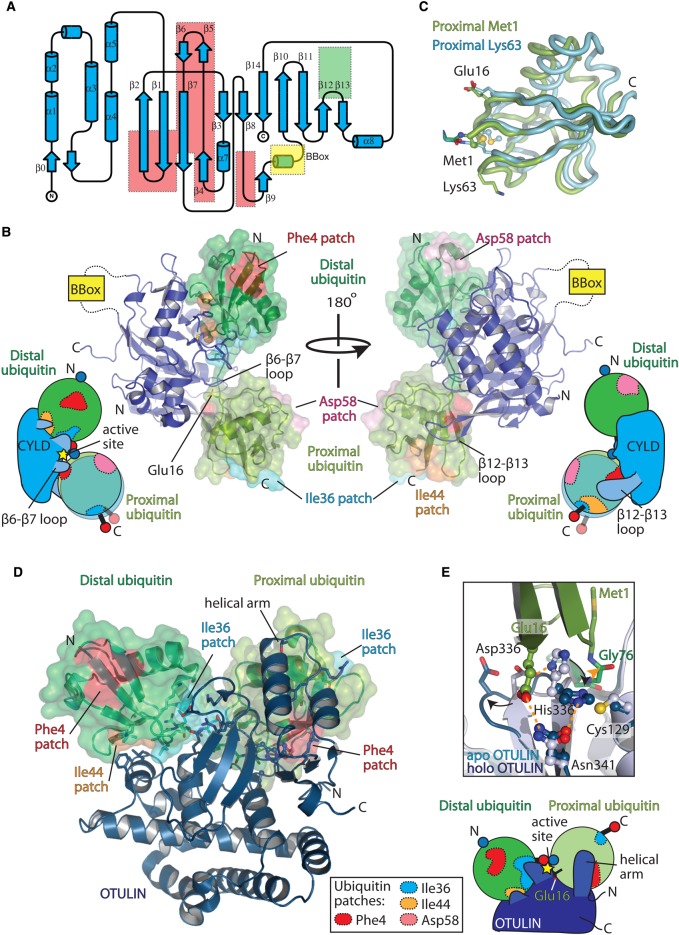
Molecular basis of Met1 disassembly (‘erasers’). (**A**) Toplogy representation (generated by TopDraw [148]) of CYLD USP domain (PDB ID: 2VHF). Regions of the USP domain that are absent from CYLD compared with other USP domains are enclosed in a red box, whereas, the β12–β13 region that contains an insertion unique to CYLD is enclosed in a green box. The region that is replaced by an inserted B-box domain is shown in yellow. (**B**) Structure of CYLD ΔB-box (blue) bound to Met1-linked diubiquitin (green surface) (PDB ID: 3WXE) is shown in two different orientations. Cartoon representation showing the binding of Lys63 and Met1 diubiquitin is shown for each orientation. The different relative positions of the proximal ubiquitin moieties for Met1- and Lys63-linked diubiquitin is shown, as are the β6–β7 and β12–β13 loops. (**C**) Superimposition of the Met1 (green) and Lys63 (blue) proximal ubiquitin moieties from the CYLD diubiquitin structures (PDB ID: 3WXE and 3WXG, respectively), highlighting the positioning of the Met1 amino-terminus and Lys63 ε-NH_2_ side chain that forms the peptide/isopeptide bond, respectively. (**D**) Structure of OTULIN (blue) bound to Met1-linked diubiquitin (green) (PDB ID: 3ZNZ). Bottom right, schematic of the OTULIN interaction with Met1-linked diubiquitin. (**E**) Zoom-in of the catalytic site of apo OTULIN (light blue, PDB ID: 3ZNV) and holo OTULIN bound to Met1-linked diubiquitin (blue, PDB ID: 3ZNZ), showing the changes that occur within the active site upon Met1-linked diubiquitin binding. Hydrogen bonds between the catalytic triad residues (His336 and Asn341) and Glu16 are shown as orange dashes. The carbonyl of Met1 diubiquitin that is attacked by the nucleophilic catalytic cysteine (Cys129) is shown by an orange triangle.

Fukai and co-workers determined the structure of zebrafish CYLD bound to either Met1- or Lys63-linked diubiquitin and demonstrated that CYLD has a selectively weakened distal (S1) site and an insertion within the proximal (S1′) binding site that allows Lys63 and Met1 specificity [100]. CYLD recognises the distal ubiquitin moiety in a different manner compared with other USPs. In part, this is due to the deletion of the canonical fingers domain (β4–β5 deleted and β6–β7 truncated; Figure 6A), resulting in a significant rotation of the distal ubiquitin moiety compared with ubiquitin-bound USP7 [100]. There are fewer contacts between the canonical Ile44 patch of ubiquitin and CYLD, with hydrophobic interactions from highly conserved residues on CYLD. Polar and electrostatic contacts from CYLD to the distal ubiquitin moiety further stabilise the association between DUB and substrate (Figure 6B). Remarkably, CYLD is capable of accommodating the Lys63 and Met1 proximal ubiquitin moieties equally well, with only a 13° rotation between them (Figure 6C). This is achieved by few, but defined, interactions to the proximal ubiquitin moiety. CYLD contains an extended β12–β13 loop, not found in other USPs, which contacts the proximal ubiquitin along the Phe4 patch and extends to the base of the Ile44 patch. Deletion of the β12–β13 loop, or a point mutation disrupting proximal ubiquitin binding, reduces activity against Lys63 and Met1 linkages without affecting the residual Lys48 activity [95,100]. The β6–β7 loop in other USPs binds to the distal ubiquitin moiety. However, in CYLD, the β6–β7 loop is truncated by five residues allowing Glu16 from the proximal ubiquitin moiety to bind.

This dynamic binding mode of CYLD is in contrast with the recognition of Met1 linkages described previously, where defined interactions to the proximal ubiquitin moiety ensure Met1-linkage specificity. Here, CYLD is capable of exploiting the topologically similar Lys63 and Met1 linkages and recognising both through binding of Glu16 from the proximal ubiquitin moiety. Furthermore, the selectively weaker distal binding site may prevent CYLD from hydrolysing monoubiquitin attached to substrates, although this remains to be conclusively investigated. This may have implications for the role of CYLD *in vivo*.

### OTU domain deubiquitinase with LINear linkage specificity

OTULIN was identified following bioinformatical predictions of unannotated OTU domain folds in two human proteins: FAM105A and FAM105B. While FAM105A does not have catalytic residues and is inactive, FAM105B readily cleaves Met1 linkages and was renamed OTULIN [91]. In parallel, OTULIN was identified from a genetic screen for neuronal phenotypes in mice [90].

The structures of OTULIN unbound and bound to Met1-linked diubiquitin revealed the molecular basis for OTULIN's specificity [90,91]. In the unbound, ‘apo’ state, the catalytic histidine, His339, is not orientated for correct deprotonation of the catalytic cysteine (Cys129), but is rather held in an autoinhibited conformation by a non-catalytic aspartate, Asp336 [90,91]. The structure of OTULIN bound to Met1-linked diubiquitin revealed extensive contacts between both distal and proximal ubiquitin moieties (Figure 6D). OTULIN binds both Ile36 and Ile44 patches of the distal ubiquitin moiety through hydrophobic interactions to the Ile44 patch and mostly polar and aliphatic side chain interactions to the Ile36 patch. Unlike CYLD, which can accommodate the proximal ubiquitin moieties from both Met1 and Lys63 linkages, OTULIN forms extensive contacts to the proximal ubiquitin moiety of Met1 linkages. OTULIN binds the Phe4 patch of the proximal ubiquitin moiety and additional interactions are made from the first two helices of the catalytic OTU domain, which form the majority of the proximal binding site. OTULIN binds Met1-linked diubiquitin with a *K*_D_ of 150 nM, 100-fold higher affinity than Lys63-linked diubiquitin [91]. More strikingly, the structure also revealed that OTULIN is directly activated by the bound diubiquitin: placement of the proximal ubiquitin inserts the ubiquitin side chain of Glu16 directly into the active site of OTULIN, thereby pushing His339 into an active conformation and additionally co-ordinating the third catalytic residue, Asn341 (Figure 6E). Consistently, mutation of the proximal ubiquitin Glu16 to alanine (E16A) reduces the catalytic efficiency (*k*_cat_) of OTULIN by 240-fold without significantly affecting binding of the substrate (similar *K*_D_ and only 3-fold reduction in *K*_M_) [91]. Only binding of Met1 diubiquitin induces the correct positioning of Glu16 into the active site, thus activating OTULIN, explaining why OTULIN is specific for Met1 linkages.

The OTULIN mechanism of ubiquitin-assisted catalysis has, to date, not been observed in other DUBs. However, conceptually similar mechanisms have been described for assembly of Lys11-linked polyubiquitin chains by UBE2S [101] and for the NEDD8 modification of SCF (Skp1, Cullin1, F-box containing) E3 ligase complexes [102].

As a result of OTULIN's mechanism of activation, it can only hydrolyse Met1 linkages but would be unable to hydrolyse the isopeptide bond between ubiquitin and substrate, unless a glutamate could be placed within the active site at the analogous position from the proximal ubiquitin moiety. Presumably, other DUBs are required to remove the remaining ubiquitin isopeptide linkage, or additionally, other E3 ligases may extend the remaining ubiquitin with a different ubiquitin chain type (‘ubiquitin chain editing’), resulting in a distinct ubiquitin signal.

## Regulation of the Met1 machinery

The intrinsic Met1 specificity in the aforementioned components is not sufficient to regulate an appropriate physiological response; time-dependent recruitment of writers, readers and erasers to defined signalling complexes is required. It is, therefore, unsurprising that the Met1 machinery is regulated through either a combination of recruitment and/or activation/inhibition mechanisms.

### Recruitment of LUBAC

LUBAC recruitment to the emerging signalling complex is essential and this is achieved through binding of HOIP to polyubiquitin chains. Elegant biochemical and *in vivo* analysis revealed that the catalytic activity of cIAPs is required for LUBAC recruitment to the TNFR complex [39]. Furthermore, in NOD2 signalling, the catalytic activity of XIAP is required for LUBAC recruitment [40]. Additionally, several groups have reported that the N-terminus of HOIP (PUB-UBA; Figure 3) binds Lys63 polyubiquitin with greater affinity than Met1 linkages [9,34,39] and that the NZF1 domain mediates this binding [34], reinforcing the notion that LUBAC binding to Lys63-linked polyubiquitin is a general mechanism for LUBAC recruitment. In addition to binding ubiquitin, the NZF1 domain is also capable of simultaneously engaging with NEMO, through non-overlapping sites [103]. Interestingly, as described above, both HOIL-1 and SHARPIN contain NZF UBDs. Furthermore, biophysical data have shown that SHARPIN preferentially binds Met1 linkages over Lys63 linkages [34].

Therefore, what is the role of Met1 ubiquitin binding by HOIL-1 and SHARPIN? No study has fully addressed this question, though one possible mechanism may result in the stabilisation/recruitment of further LUBAC complexes to the receptor complex, resulting in an amplification of Met1 linkages, and thus facilitating further NEMO recruitment and IKK activation.

### Activation of LUBAC

**Figure 7.**
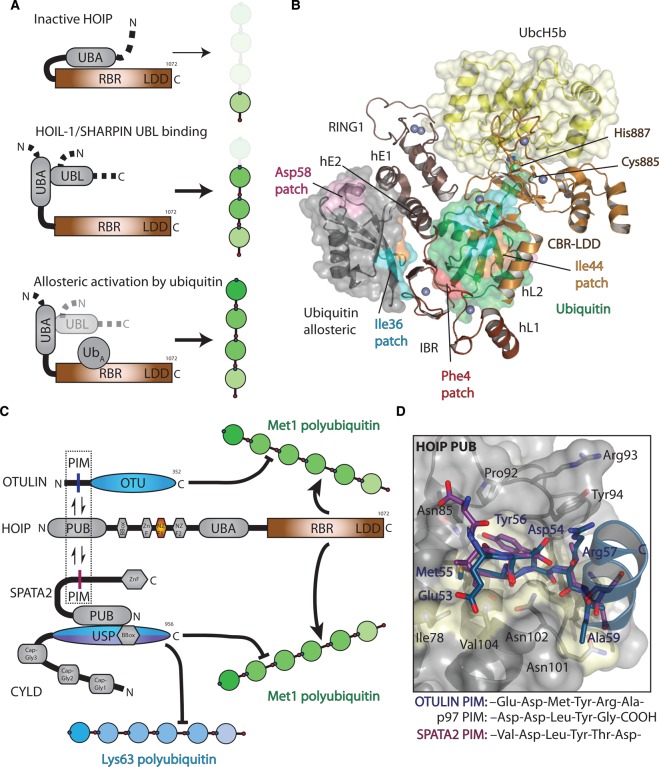
Regulation of the Met1 signal. (**A**) Top, HOIP is autoinhibited through suspected binding of its UBA domain to the RBR domain, preventing Met1-linked polyubiquitin synthesis. Middle, binding of either HOIL-1 or SHARPIN UBL domains to the HOIP UBA domain releases autoinhibition and activates HOIP, allowing generation of Met1-linked polyubiquitin chains. Bottom, in addition to HOIL-1 or SHARPIN binding, the HOIP RBR domain contains an allosteric ubiquitin-binding site (Ub_A_) that can also activate Met1 polyubiquitin formation. (**B**) Structure of the HOIP RBR bound to E2 (UbcH5b)∼ubiquitin (yellow and green surface) and non-covalent ubiquitin (ubiquitin allosteric, grey surface; PDB ID: 5DEV). Different hydrophobic patches are shown on both ubiquitin surfaces, showing the extensive interactions between the RBR and bound ubiquitin. (**C**) The activity of LUBAC is further regulated through DUB (OTULIN and CYLD) binding to the HOIP PUB domain. OTULIN contains an internal PIM that allows it to bind to the HOIP PUB domain. However, CYLD does not contain a PIM but is bound to SPATA2, which contains a PUB domain that specifically recognises the CYLD USP domain. SPATA2 also contains an internal PIM that allows it to bind to HOIP in an identical way as OTULIN. (**D**) Structure of the OTULIN PIM (PDB ID: 4OYK, blue) bound to the HOIP PUB domain (grey surface with interacting residues coloured yellow) and the SPATA2 PIM (PDB ID: 5LJN, purple). For clarity, only the OTULIN residues are annotated in blue. The PIM sequences that are able to bind the HOIP PUB domain are shown below for OTULIN, p97, and SPATA2.

HOIP is autoinhibited by its UBA domain through unknown mechanisms. Binding of either HOIL-1 or SHARPIN is capable of relieving this autoinhibition [50,51] (Figure 7A). Interestingly, although no significant HOIL-1 E3 ubiquitin ligase activity could be detected *in vitro* [50,51], binding of full-length HOIL-1 to HOIP not only relieved autoinhibition of HOIP, but also resulted in an increase in Met1-chain production compared with activation by either SHARPIN binding or HOIL-1 UBL binding [51]. Furthermore, the *in vitro* ubiquitination of NEMO requires the active site cysteine of HOIL-1 (Cys460), in addition to HOIP, and serves to direct polyubiquitination onto NEMO [104], suggesting an association between the HOIP and HOIL-1 RBR domains, through a mechanism that remains to be determined. Parallels could be drawn to a recent study from Schulman and co-workers, who have identified a two-step chain formation by an RBR (human homologue of ariadne (HHARI)/ARIH1) and a cullin ring ligase (CRL) [105]. The autoinhibition of ARIH1 is relieved upon association with the NEDD8-modified CRL. Activated ARIH1 then adds the first ubiquitin onto a substrate followed by polyubiquitin chain assembly by the CRL [105]. Clearly, more work is required to understand the additional functions of HOIL-1.

The structure of the HOIP RBR bound to charged E2 and ubiquitin unexpectedly revealed an additional ubiquitin-binding site within the RBR domain on the opposite face of the RING1–IBR (in-between RING) linker helices [49] (Figure 7B). Hydrophobic interactions are formed from the linker helix (hE1) to the C-terminus of ubiquitin, with additional electrostatic interactions from a β-hairpin in the IBR domain. Binding studies revealed that diubiquitin binding enhances subsequent binding of E2∼ubiquitin and further chain formation. The equivalent linker helix in HHARI and Parkin structures is kinked [106–108]. Intriguingly, in a recent structure of Parkin bound to phospho-ubiquitin, binding of phospho-ubiquitin to this site results in a straightening of the linker helix and reorientation of the RING1–IBR subdomains [109]. It still remains to be shown whether such a helix straightening occurs within the HOIP RBR upon ubiquitin binding and whether this is required for true activation of HOIP.

Mouse knockout studies have implied that HOIL-1 and SHARPIN form an integral part of LUBAC *in vivo*, since loss of either HOIL-1 or SHARPIN results in a reduction in HOIP levels [33–35]. This suggests that HOIP probably never exists alone and is always in complex with either HOIL-1 or SHARPIN. As described above, either binding of HOIL-1 or SHARPIN is sufficient to activate HOIP. Therefore, what would prevent LUBAC from generating superfluous Met1 polyubiquitin chains, resulting in the recruitment of NEMO and unwanted NF-κB activation?

### LUBAC–DUB interactions

The above question was answered by the discovery that OTULIN and CYLD interact with LUBAC [110–112] (Figure 7C) and further consolidated the role of OTULIN as a *bona fide* regulator of Met1 signalling. An interaction between HOIP and OTULIN or CYLD at first inspection would seem contradictory, as a futile energy-consuming cycle would exist. However, E3 ligases are commonly found bound to DUBs [113] and have defined roles in ubiquitin chain editing or DUB/E3 ligase stability [114–116].

Mass spectrometry and biochemical analysis showed that OTULIN binds to the HOIP PUB (peptide: N-glycanase/UBA- or UBX-containing proteins) domain [110,111]. The N-terminus of OTULIN contains a PUB interaction motif (PIM) sequence: Asp54–Met55–Tyr56–Arg57–Ala58, which is highly conserved across OTULIN orthologues. The OTULIN PIM binds HOIP with 40-fold greater affinity than a similar PIM from p97. Moreover, the OTULIN PIM is specific for HOIP in that it does not bind PUB domains from PNGase or UBXN6 [110]. Structures of the HOIP PUB domain in complex with peptides from OTULIN or p97 revealed the molecular basis for this interaction and PUB domain specificity [110,111] (Figure 7D): HOIP interacts with residues from the OTULIN PIM not found in the p97 PIM and is unable to interact with the free C-terminus of p97. This is in contrast with PNGase, which engages a salt-bridge interaction from the PUB domain to the free C-terminus of p97 [117].

However, CYLD does not contain a PIM and although several studies had shown a dependence on a functional PIM-binding site in the HOIP PUB domain [42,112,118], no direct interaction between CYLD and the HOIP PUB domain could be observed *in vitro*. Recently, four independent studies have identified a previously uncharacterised protein, spermatogenesis-associated factor 2 (SPATA2), as a critical component that links CYLD to HOIP [119–122]. SPATA2 and SPATA2L had been previously identified in a DUB-wide proteomics screen as a strong interactor with CYLD [113]. However, the implications of such an interaction have only recently been investigated. SPATA2 contains a PUB domain but, unlike the HOIP PUB domain, it does not recognise canonical PIMs. Instead, the SPATA2 PUB domain binds strongly (*K*_D_ 10 nM) to the CYLD USP domain and the interaction is strengthened through dimerisation of CYLD, mediated via its B-box domain [122]. In addition, SPATA2 binding activates the catalytic activity of CYLD by 2-fold. Furthermore, SPATA2 contains an internal PIM that binds to the HOIP PUB domain, which is crucial for CYLD recruitment to the TNFR complex (Figure 7C).

Interestingly, OTULIN and SPATA2 are not found in the same ligase complex together, suggesting competition for the HOIP PUB domain. Even though SPATA2 and OTULIN PIMs have similar affinities and binding modes to the HOIP PUB domain [122] (Figure 7D), avidity effects, through CYLD dimerisation presenting two SPATA2 PIMs to a multimeric LUBAC complex, may simply outcompete OTULIN binding.

Additionally, either the DUB or LUBAC may undergo post-translational modifications to regulate the association. In the case of PNGase–p97 interaction, phosphorylation of the conserved Tyr805 in p97 prevents binding to PNGase [117]. Consistently, phosphorylation of the equivalent residue in OTULIN abolishes the interaction with HOIP [110]. Tyr56 in OTULIN has been identified as a site for phosphorylation [110]. However, the kinases or phosphatases regulating this currently remain unknown. Likewise, it is unclear whether the SPATA2 PIM can be phosphorylated to regulate association with LUBAC.

Recent reports have suggested different roles of OTULIN–LUBAC and CYLD–LUBAC complexes *in vivo*. OTULIN prevents Met1-linked autoubiquitination of LUBAC as knockdown of OTULIN results in elevated Met1 linkages on LUBAC [118,123], whereas CYLD does not regulate the levels of autoubiquitination on LUBAC [42,118]. Draber et al. [118] have further suggested that OTULIN is not recruited to the TNFR1 or NOD2 signalling complexes in contrast with other studies [111,123]. However, the NOD2 signalling complexes are regulated by OTULIN, regardless of OTULIN's recruitment [42]. Clearly, more work is required to delineate the roles of OTULIN and CYLD in inflammatory signalling and this may depend not only on the type of signalling complexes (for example, TNFR1, NOD2, or IL-1β), but also on the timing following receptor activation and the dependence on additional factors, such as SPATA2. Recently, patients with mutations within the *OTULIN* gene have been identified. These patients present a potentially fatal autoinflammatory disorder [124,125]. This has been further validated in mouse model studies where an increase in Met1 chain formation and autonomous NF-κB signalling are observed [125].

### DUB regulations

The mechanism of ubiquitin-assisted catalysis described earlier for OTULIN represents one way in which a DUB can be regulated to ensure the correct substrate is cleaved once presented. In addition, post-translational modifications can also regulate DUB activity. The most noted example is the phosphorylation-dependent activation of OTUD5 [126]. However, other types of DUB modification exist and have been reviewed [127].

CYLD can also be regulated by phosphorylation, and this occurs outside the catalytic USP domain. The exact mechanism of CYLD phosphorylation remains to be investigated since two reports, [128] and [129], suggest that phosphorylation either inhibits or activates CYLD, respectively. Recently, CYLD has also been shown to undergo modification by the ubiquitin-like modifier SUMO in response to all-*trans*-retinoic acid treatment of neuroblastoma cells. SUMOylation of CYLD occurs at residue Lys40 in the N-terminus of CYLD and although it is separate from the USP domain, it is capable of inhibiting DUB activity [130]. Finally, caspase-8 has been shown to cleave CYLD immediately after the first CAP-Gly domain, which results in subsequent CYLD degradation via the proteasome. Cleavage of CYLD results in pro-survival signals through the prevention of necroptosis [131].

One long held conundrum has been the different *in vitro* specificity of purified recombinant A20 versus A20 *in vivo* (Lys48 versus Lys63, respectively) [78,81]. Recently, it has been shown that phosphorylation of A20 appears to alter linkage specificity, in favour of Lys63 linkages, explaining the aforementioned discrepancy [79]. It will be of interest to understand the molecular mechanisms by which phosphorylation of the catalytic OTU domain of A20 alters linkage specificity.

### Regulation of Met1 UBDs

LUBAC is capable of ubiquitinating the NEMO CoZi domain at positions Lys285 and Lys309 [31,132]. A recent study using an *in vitro* reconstituted IKK assay has demonstrated that ubiquitinated NEMO activates IKK more than unanchored Met1 linkages [103]. Additionally, the same group showed that HOIP NZF1 domain interacts directly with NEMO. Interestingly, HOIP NZF1 does not bind to the CoZi site and the NZF1 domain is capable of binding to NEMO and ubiquitin simultaneously. The attachment of Met1 diubiquitin onto NEMO might be sufficient to induce the conformational change described in the previous section: Nuclear factor-κB essential modifier. However, the roles of Met1-ubiquitinated NEMO in IKK activation are currently unclear since other studies have not identified Met1 linkages on NEMO in IL-1β-stimulated cells [9], and no study has demonstrated the induction of Met1 linkages on NEMO during receptor stimulation or whether only a small subset of NEMO contains Met1 linkages. Another model would be that ubiquitinated NEMO serves to recruit other NEMO molecules in *trans*, resulting in IKK clustering and concurrent activation [103]. Additionally, the ZnF domain of NEMO interacts with IκBα and brings the substrate to the IKK complex [133]. Clearly, the mechanisms of IKK activation warrant further investigation.

In addition to potentially activating NEMO, other ligases have been shown to ubiquitinate NEMO and regulate its function: Trim23 ubiquitinates positions Lys165, Lys309, Lys325, and Lys326 during antiviral defence signalling that does not result in NF-κB activation [134].

A20 is also recruited to the inflammatory signalling complexes, although this is not via a direct interaction with LUBAC but through the association of ZnF7 with Met1 polyubiquitin chains [84]. A20 appears to stabilise Met1 linkages in TNFR1 and NOD2 signalling complexes through the binding of ZnF7 preventing DUB cleavage but also competing for NEMO recruitment, reducing NF-κB activation [118]. Since *A20* gene induction is driven by NF-κB, the accumulation of A20 will induce a negative feedback loop to the inflammatory signalling complexes, although such a model would require further validation through *in vitro* and *in vivo* competition experiments and quantification of protein recruitment to the stimulated receptor complex. The latter experiments are technically challenging owing to heterogeneity between cell receptor expression levels and asynchronous activation of receptors.

### Proteolytic cleavage of Met1 regulators

In addition to CYLD cleavage by caspase-8 described earlier, proteolytic cleavage of several regulators of the Met1 machinery has been reported, notably in B- and T-cell receptor signalling. Although the functions of Met1-linked polyubiquitin linkages in B- and T-cell signalling are enigmatic, functional roles of LUBAC are emerging [45,46,135]. Antigen receptor stimulation results in the assembly of the molecular scaffold and paracaspase CBM signalosome (reviewed in ref. [136]). The CBM complex serves to activate NF-κB and JNK signalling pathways through the recruitment of the IKK and TAK1 kinase complexes. Additionally, the paracaspase activity of MALT1 results in the cleavage of several negative regulators of NF-κB and JNK signalling, notably A20 [137,138] and CYLD [139]. Recently, three separate studies have identified HOIL-1 as a substrate for MALT1 [140–142]. The functional relevance of HOIL-1 cleavage warrants further investigation, since the N-terminus of HOIL-1 remains associated with HOIP, whereas the C-terminal ZNF-RBR domains are no longer recruited to the CBM and may mediate alternate functions [140–142]. It will be interesting to see whether the co-operative role of HOIL-1 in directing Met1 linkages onto substrates, as described in the *Activation of LUBAC*, is playing a role in CBM signalling. Interestingly, in a recent mouse knockout study, loss of OTULIN in B- or T-cells results in no overt phenotype as the levels of HOIP and SHARPIN are strongly reduced while, curiously, the levels of HOIL-1 remain unaffected [125].

### The emerging roles of branched chains

Until recently, most descriptions of ubiquitin within inflammatory signalling complexes have focused on homotypic polyubiquitin linkages. For example, the importance of Lys63 chains for the TAB1–TAK1–TAB2/3 kinase complex activation and Met1 chains for IKK recruitment and activation has been shown; see [25] and [19,60,62–64], respectively. However, TAK1 is required for the *in vivo* activation of IKK [71]. Additionally, TAK1 kinase complex phosphorylates IKKβ upon binding of TAB2/TAB3 to Lys63 polyubiquitin chains [25]. A recent study has delineated the sequence of events of IKK activation [143], whereby phosphorylation of IKKβ Ser177 is required before IKKβ autophosphorylation. Furthermore, IKKβ autophosphorylation is suppressed in the Met1 binding-deficient NEMO mutant D311N, suggesting that IKK binding to Met1 chains is needed for TAK1 phosphorylation [143].

The identification of branched Lys63/Met1 chains in IL-1β signalling conjugated to the proximal receptor kinases, interleukin-1 receptor-associated kinase 1 (IRAK1) and IRAK4, and the adaptor MyD88 [9], provides an elegant solution to the activation of the IKK complex through both Lys63 and Met1 linkages. Additionally, the roles of branched Lys63/Met1 chains are emerging in other inflammatory signalling pathways such as the NOD2 receptor complex [42,144], suggesting that this could be a unifying mechanism for the TAK1-dependent activation of the IKK complex and explaining the differing and important roles of Lys63 and Met1 polyubiquitin linkages.

## Conclusions and future perspectives

Investigations into the enzymes and proteins that control the Met1-linked polyubiquitin signal have interrogated another strand of inflammatory signalling. Understanding the mechanisms of specificity at the molecular level has not only provided insights into Met1-linked polyubiquitination and inflammatory signalling, but has also elucidated new and novel mechanisms of protein regulation and enzyme activation. Such a molecular understanding allows the design of precise point mutations, which can subsequently be introduced into cells and model organisms. This approach, as opposed to designing clumsy domain deletions that all too often affect protein stability or other binding events, enables careful dissection of the Met1-linked polyubiquitin signal.

The next challenge will be to understand which defined signalling complexes are recruited upon inflammatory signalling. This is exemplified by the lack of a stable OTULIN complex at the TNFR1 signalling complex, despite OTULIN being clearly important for regulating Met1-linked polyubiquitin chains. The recent identification of SPATA2 as a missing link between HOIP and CYLD provides a new component to investigate the competition of OTULIN and also generates insights into CYLD regulation. Importantly, all of the components involved in regulating the Met1 signal are themselves regulated, often by other post-translational modifications, providing another layer of control. Investigating these regulatory mechanisms will provide further insights into the Met1 pathway and also will help understand how defects in the regulation often lead to disease.

As the protein interaction network of components in the Met1 polyubiquitin signal expands, and new interactors are identified, the question becomes: are Met1-linked polyubiquitin chains involved in other pathways? For example, are there roles of Met1-linked polyubiquitin chains in mitophagy? Since the E3 ubiquitin ligase Parkin has been shown to bind HOIP [145] and Parkin contains a UBL domain (like in HOIL-1 and SHARPIN). Likewise, OTULIN has been linked to Wnt signalling [90], suggesting roles of Met1 linkages in the Wnt pathway. SHARPIN, on the other hand, has been shown to control β-integrins, independent of Met1-linked polyubiquitin [146,147]. Therefore, SHARPIN may have other roles outside of LUBAC activation.

Finally, what are the roles of branched polyubiquitin chains, in particular Lys63/Met1 branched chains? Do defined readers exist that detect branched linkages? Or do they function to scaffold and recruit other signalling components into close proximity for enhanced activation?

Certainly, the next 10 years of research into the Met1-linked polyubiquitin signal will yield many more exciting discoveries.

## Abbreviations

CAP-Gly: cytoskeletal-associated protein-glycine-conserved
CBM: CARMA1–BCL10–MALT1
CC: coiled coil
cIAP1/2: cellular inhibitor of apoptosis 1/2
CRL: cullin ring ligase
CYLD: cylindromatosis tumour suppressor
DUB: deubiquitinating enzyme
HECT: homologous to E6-AP C-terminus
HOIL-1: haem-oxidised IRP2 ubiquitin ligase-1
HOIP: HOIL-1-interacting protein
IBR: in-between RING
IKK: IκB kinase
IRP2: iron-responsive element-binding protein 2
IL-1β: interleukin-1β
IRAK1: interleukin-1 receptor-associated kinase
IRAK4: IL-1 receptor-associated kinase 4
IκB: inhibitor of κB
LDD: linear ubiquitin chain determination domain
LUBAC: linear ubiquitin chain assembly complex
LZ: leucine zipper
Met1: methionine1
MyD88: myeloid differentiation primary response gene 88
NEMO: nuclear factor-κB essential modifier
NF-κB: nuclear factor-κB
NOD1/2: nucleotide and oligomerisation domain 1/2
NZF: nuclear protein localisation 4 (Npl4) zinc finger
OTU: ovarian tumour
OTULIN: OTU domain deubiquitinase with a LINear linkage
PIM: PUB interaction motif
PUB: peptide: *N*-glycanase/UBA- or UBX-containing proteins
RBR: RING in-between RING
RING: really interesting new gene
RIPK1: receptor-interacting protein kinase 1
SHARPIN: SHANK-associated RH domain interactor
SPATA2: spermatogenesis-associated factor 2
TAB: transforming growth factor-β-activated kinase 1-binding protein
TAK1: TAB-activated kinase 1
TNF: tumour necrosis factor
TNFR1: TNF receptor 1
TRAF6: TNF receptor-associated factor 6
UBD: ubiquitin-binding domain
UBZ: ubiquitin-binding zinc (Zn) finger
USP: ubiquitin-specific protease
XIAP: X-linked inhibitor of apoptosis protein
ZnF: Zn finger
